# Evaluation and Analysis of Push-Pull Cable Actuation System Used for Powered Orthoses

**DOI:** 10.3389/frobt.2018.00105

**Published:** 2018-09-11

**Authors:** Svetlana Grosu, Carlos Rodriguez–Guerrero, Victor Grosu, Bram Vanderborght, Dirk Lefeber

**Affiliations:** MECH Department, Vrije Universiteit Brussel (VUB) and Flanders Make, Brussels, Belgium

**Keywords:** push-pull cable, cable-conduit, exoskeleton, rehabilitation robotics, cable-based actuation

## Abstract

Cable-based actuation systems are preferred in rehabilitation robotics due to their adequate force transmission and the possibility of safely locating the motors away from the patient. In such applications, the cable dynamics represents the prescribing component for the system operating loads and control. A good understanding of the actuation, based on cable-conduit transmission, is therefore becoming mandatory. There are several types of cable-conduit configurations used for the actuation. Currently, there is lack of information in literature with regard to the push-pull cable type. Therefore, the main focus of this contribution is to evaluate push-pull cable-based actuation used within wearable robotic devices. This study includes working principle description of push-pull cable actuation with its characteristic advantages and drawbacks. The use of push-pull cables in bidirectional force transfer with remote actuation is investigated being integrated in a test-stand setup of a novel gait rehabilitation device. The experimental results and close analysis of the push-pull cable-based actuation system outline its performance, the overall dynamic behavior and the transmission efficiency of push-pull cables used for powered orthoses.

## 1. Introduction

In the development of novel rehabilitation robotic devices engineers are faced with the challenge of combining suitable design concepts, high performance actuator technologies and dedicated control strategies in view of improved physical human-robot interaction (HRI). According to a number of investigations on different actuation approaches for exoskeletons, the low power/weight and force/angle ratios are still major drawbacks (Herr, 2009). Classical designs including high power actuators tend to be relatively expensive. Typically these are bulky, heavy, and have a high mechanical output impedance due to necessary power transmission. In addition, actuators directly integrated on the joints would add unnecessary weight to the orthoses. In order to compensate for their own weight, the size of the motors must increase, escalating the required power from joint to joint. This will conduct to a significant increase in total system mass and inertia. The solution suggested in several contributions (Morrell and Salisbury, 1998; Sugar, 2002; Zinn et al., 2004; Veneman, 2006; Slavnić et al., 2014; Guerrero et al., 2015) proposes relocating all actuators to the static base of the system and decoupling the dynamics of the actuator and the load, by using a compliant element, e.g., a spring, between both. This way, mass and inertia of the movable part can be significantly reduced, thus, allowing an ergonomic kinematic design.

Cable driven actuation, such as: open-ended cables (Tsai, 1994), endless cables, Bowden cables, and push-pull cables are a promising alternative when a combination of lightweight, high strength, compact designs, safety, compliance, and dynamic motions are required. Generally, the torque capacity of cable-based actuators is a function of the strength of the cable. As well, the efficiency of cable drives can reach up to 96%, on condition that they are properly implemented (Townsend and Salisbury, 1988). Initially, open-ended cables were used in actuation of robotic devices, but they were limited to providing only tension force and no compression force, so that an extra device was needed to hold the cables in tension, complicating the design and the control of the transmission system. To overcome this problem, a new generation of cables was developed: endless tendon drives (Tsai, 1994), Bowden cables (Veneman et al., 2005; Sulzer et al., 2009), and push-pull cables (PPC) (Winter and Bouzit, 2007; Grosu et al., 2014; Slavnić et al., 2014; Xu et al., 2014; Guerrero et al., 2015).

In recent years, cable-conduit actuation gained significant attention in rehabilitation robotics (Springer and Ferrier, 2002; Wege and Hommel, 2005; Veneman, 2006; Dovat et al., 2008; Sulzer et al., 2009; Slavnic et al., 2013; Wu et al., 2015), mainly due to advancements in high-strength cable materials which support the transmission of high forces and offer the possibility of locating actuators away from the patient. However, these advantages are overshadowed by the nonlinear dynamic behavior caused by friction between the cable and the conduit. Backlash effect is more evident due to compliance and friction within the conduit when the actuation direction is changed. This issue can result in a loss of precision and has to be compensated for in control algorithms. As an example of this approach, an adaptive backlash inverse controller was developed by Agarwal et al. (2010) that dynamically estimates the model parameters and compensates for changes in friction influenced by the conduit curvature. These drawbacks were reported also in various studies where cable actuation is used (Townsend and Salisbury, 1988; Kaneko et al., 1991; Panchaphongsaphak et al., 2006; Agrawal and Peine, 2008). The backlash effect produced by using cable-driven mechanisms on surgical robot was evaluated in Peine et al. (2012). Some researchers have investigated means of reducing the coefficient of friction in cable and housing systems (Sammons, 1983; Carlson et al., 1990). LeBlanc (1990) showed efficiency depends upon the angle of wrap, the types of the cable and housing used. The effect of friction coefficient and other variables on frictional losses in upper-limb prostheses was researched in Carlson et al. (1995). The following parameters of interest were investigated: type of the cable and cable housing; the angle through which the cable bends; bending radius and the amount of tension in the cable. Various techniques are adopted in practice to reduce the friction effects, e.g., by using PTFE-coated steel cables and keeping wide angles for cable-wrapping (Letier et al., 2006). But, these hardware-based solutions can reduce the friction levels only to a certain degree. The other way to deal with cable-conduit nonlinear dynamics is to implement effective controllers (Agarwal et al., 2010; Vitiello et al., 2013; Slavnić et al., 2014; Guerrero et al., 2015) where the control parameters must be adjusted to the certain configuration of the cable (Panchaphongsaphak et al., 2006).

From the available cable-driven solutions further in this work we propose to focus on push-pull cable actuation. This paper provides a detailed description of general PPC technical specifications. The main goal is to investigate the transmission efficiency, mechanical design and implementation of PPC actuation system into exoskeletons. The authors describe issues related to working principles, geometric, kinematics, and dynamics particularities of the PPC actuation system.

Another goal was to estimate the motor torque τ_*m*_ in the experimental setup in conditions that only force sensors are available.

Following section 1 where the state-of-the-art of cable-based transmission systems was presented, section 2 will describe the materials and methods. Here, the working principle, transmission efficiency, and friction characteristics of the PPC are discussed. The description of experiments using an orthosis test setup which is a simplified version of a novel gait rehabilitation device CORBYS, can be found at the end of the section. The experimental results of the PPC actuation system are presented in section 3. In sections 4 and 5 the authors present main observations, results, and conclusions of the complete work.

## 2. Materials and methods

### 2.1. Physical characteristics of push-pull cable transmissions

**Figure 1:**
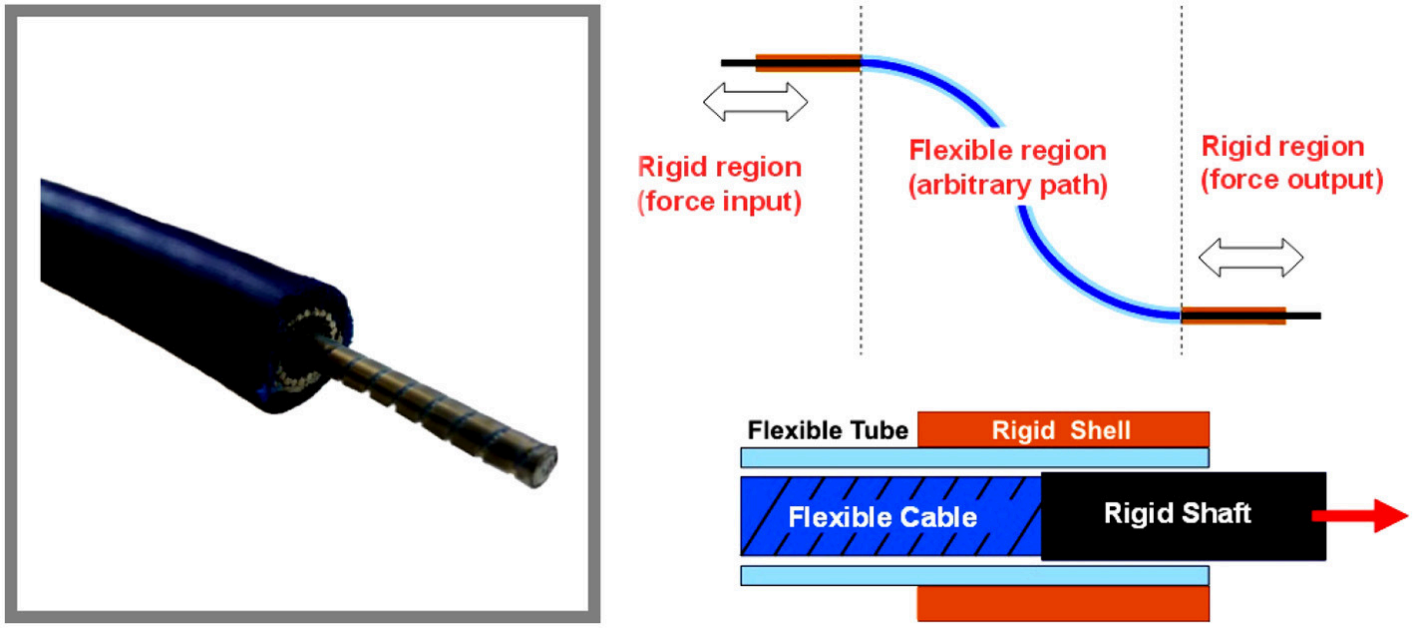
General structure of push-pull control cable configuration.

Push-pull control assemblies are designed to provide smooth, positive, and precise transmission of the mechanical motion for medium to heavy-duty push-pull applications. The general structure of a PPC consists of an inner member, made from a wire rope and armored with a polished flat band wrap covered with an inner tube. The external layer is represented by an extruded plastic mantle of great strength and durability, see Figure 1. The inner member can easily slide in low friction lifetime lubricant. Also, the end borders of the cable are featured with stainless materials and seals, ensuring protection against foreign matter and corrosion.

The main parameters influencing the cable performance are the normal forces on the cable determined by cable tension or preload, the friction coefficients resulting from material combinations and velocity of the inner member. Furthermore, cable and conduit stiffnesses play an important role in the definition of stick-slip behavior and consequently, the mechanical bandwidth of the transmission.

As mentioned before, friction between the inner member and the external conduit usually has an impact on the assembly efficiency. This is also the case for durability and control where the friction factor depends upon the total degrees of bending in the cable. The friction can be expressed by the equation, described in Schiele et al. (2006):

(1) $$\begin{array}{l} \frac{F_{in}}{F_{out}}=e^{-\mu \theta } \end{array}$$

where *F*_*in*_/*F*_*out*_ is the ratio of input to output forces, μ is the kinetic coefficient of friction and θ is the total wrap angle.

**Figure 2:**
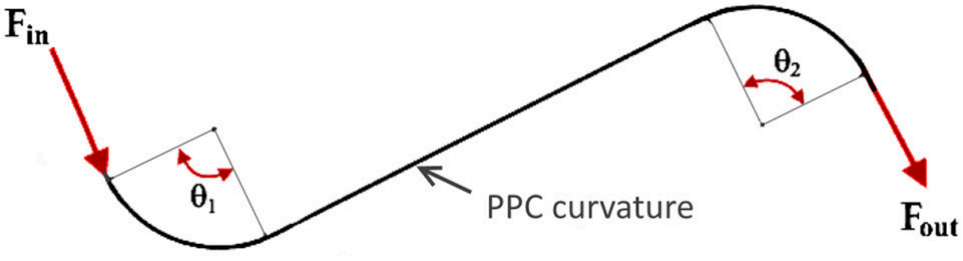
Total wrap angle estimation in the push-pull cable.

The total wrap angle (θ) of the cable system is represented in Figure 2 and defined in the equation:

(2) $$\begin{array}{l} \theta =\theta_{1}+\theta_{2}=\overset{n}{\underset{i=1}{\sum }}\theta_{i} \end{array}$$

This is why cable manufacturers advise keeping the push-pull cables as straight as possible in a setup.

**Figure 3:**
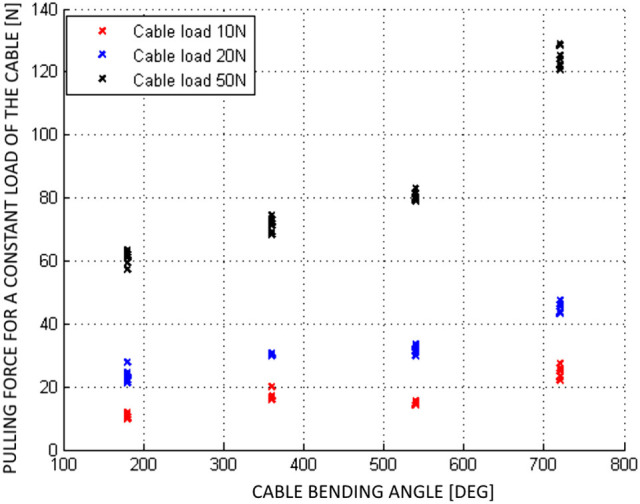
Static friction experimental results of the push-pull cable for different loads (Slavnić et al., 2014).

There is only limited literature on experimental evaluation of friction between the cable and outer conduit for push-pull cables. The experimental results on PPC static friction evaluation were presented in Slavnić et al. (2014), with the focus on the dependence of static friction on the bending angle while using different cable loads. For these experiments the PPC of 2 m length has been considered, while the bending angle of the cable was set to 180, 360, 540, and 720°. The actuation pulling force was exerted on one end of the cable and recorded at the moment when the other end of the cable started the movement. In Figure 3 can be observed that the effectiveness of the PPC for bending angles between 180° and 720° is in the range of ≈ 85–40%. While the maximal efficiency is achievable if the cable is mentioned straight and it goes up to ≈95%. Furthermore, according to PPC cables manufacturers the efficiency factor may vary due to length, strike, movement direction, bend radius and temperature. In this sense cable features such as structural modifications, cable size, end connectors types and cable lengths should be adapted to the design requirements. The selection of the proper push-pull cable, generally, is a function of the desired input force. However, in a real cable-conduit based setup, changing the bending angle modifies the cable preload and therefore also has a fundamental effect on the cable efficiency. Consequently, the cable preload increases the amount of friction as the normal forces between the cable and external conduit get bigger.

### 2.2. Test-stand mechanical design

**Figure 4:**
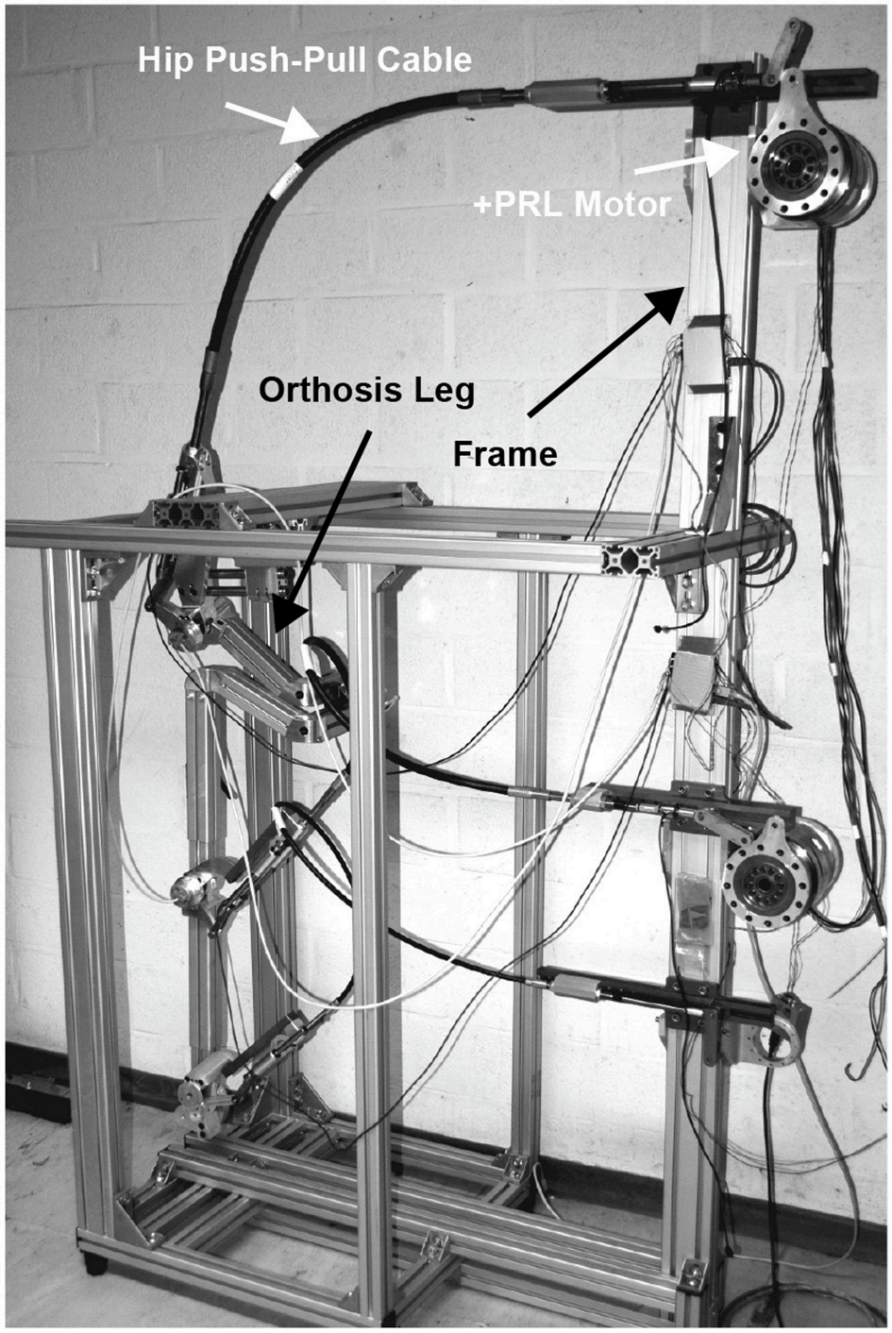
CORBYS test-stand design representation.

In the initial testing phase and the evaluation process of PPC-based actuation system, a test-stand setup of the CORBYS gait rehabilitation device (Slavnić et al., 2014) was built by project partners from SCHUNK. This test-stand was meant to prove the actuation system design concept through various experiments, as well as supporting development of the sensor processing and control algorithms to be used in the final CORBYS system prototype. The test-stand device, see Figure 4 consists of an orthosis leg and PPC-based actuation system. The frame is supporting three PRL+ motors that actuate the orthosis joints in the sagittal plane. The orthosis leg includes three revolute joints: at the ankle, knee, and hip. The mass of the orthosis is 9.2 kg. Three push-pull cable elements are connected between the actuators and joints of the orthosis leg. The selected PPCs have different geometry properties (diameters, lengths etc.) since different moments are required for the three joints actuation.

There are two U9C force cells implemented on extremities of each PPC: one force cell is located close to the actuator, the second one is on the joint side.

In Table 1 are presented the general technical specifications of the PPCs used for CORBYS test-bed actuation system. [t]

**Table 1:** Push-pull cables technical specifications, integrated in the test-stand device.

**Leg**	**Outer cable**	**Stroke**	**Cable**	**Min bend**	**Max push**	**Max pull**
**joint**	**size, mm**	**mm**	**length, cm**	**radius, mm**	**load, N**	**load, N**
Hip	17.6	152	130	153	1,350	4,500
Knee	13.3	102	130	76	450	1,035
Ankle	8.8	102	150	51	270	540

### 2.3. Working principle of PPC actuation system

In contrast to Bowden cables, that can transfer force only in pulling direction, PPCs are bi-directional, able to transfer force in pulling and pushing directions. The PPC cables are therefore larger in diameter, stiffer, and able to transmit larger forces.

**Figure 5:**
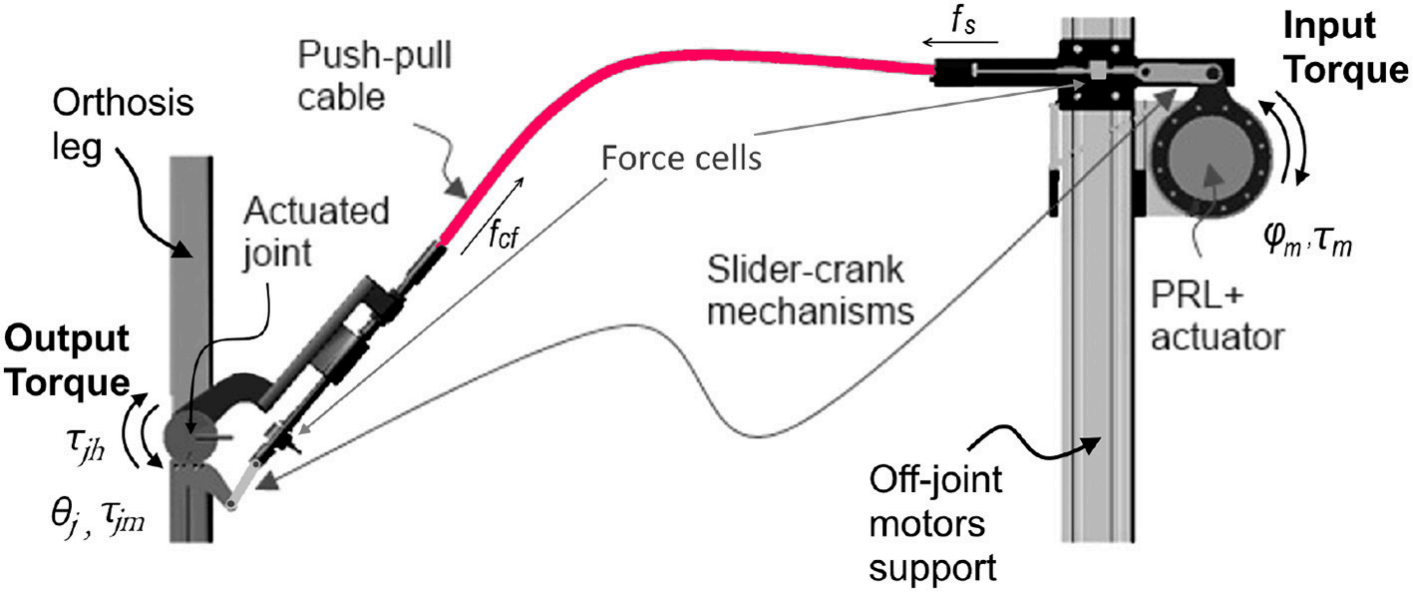
Working principle of push-pull cable, CAD respresentation of a single joint CORBYS actuation system.

Figure 5 shows one of the actuated orthosis joints with the relative kinematic and dynamic variables. In order to transmit rotational motion from motors to the orthosis joints via PPC cables, rotational motion of the motor has to be transformed into linear displacement. Then, on the joint side the linear displacement of the PPC has to be transformed to a rotational motion. For this reason, mechanically simple slider-crank mechanisms are used. The motor generates a moment τ_*m*_ that is converted to the cable force *f*_*s*_ by the slider-crank mechanism. This force is subsequently transmitted to the orthosis joint via PPC cables. The overall efficiency of the PPC actuation system is determined by the efficiencies of the sub-systems components: the efficiency of the PRL+ motor, the efficiency of the slider-crank mechanisms and the efficiency of the PPC.

**Figure 6:**
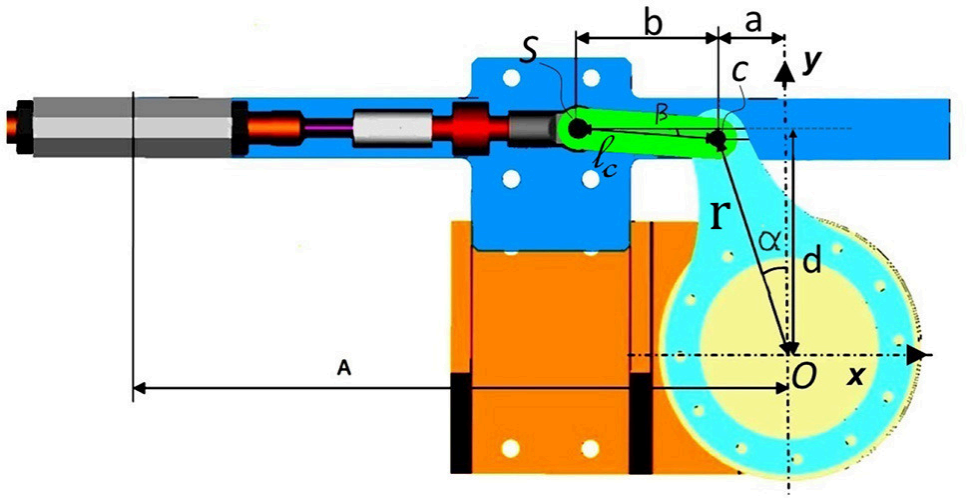
Slider-crank mechanism representation displaced on motor side.

Figure 6 shows the slider-crank mechanism displaced on the motor side of the PPC actuation system. The length of the crank is denoted by *r*, while *l*_*s*_ is the length of the connecting rod. The angular displacement of the crank is represented by α and *d* is the normal displacement between the crank pivot point and the slider line.

The crank pivot point is marked with *O* where the coordinate x-y system is located. The angle that the connecting rod makes with the slider line is denoted as β. With point *C* we indicate the revolute joint between the crank and the connecting rod. *S* marks the revolute joint between the connecting rod and the slider. Position of point *S* according to x-y coordinate system is expressed as following:

(3) S=[xsys]=[-r sin α-ls cos βr cos α+ls sin β]=[xsd]

The angle β and angular velocity $\dot{\beta }$ can be calculated using the following expressions:

(4) β= sin -1(r cos α-dls)

(5) β˙=−r sin αls cos β α˙

The lengths of the connecting *l*_*s*_ and crank rods *r* are different for the slider-crank mechanisms displaced on the orthosis joints and on the motor side, as can be seen in Table 2.

**Table 2:** Technical specifications for slider-crank mechanisms.

**Parameter**	**Units**	**Hip orthosis**	**Hip motor**	**Knee orthosis**	**Knee motor**
Crank length (*r*)	[mm]	100	110	90	90
Connecting rod (*l*_*s*_)	[mm]	80	70	60	70

The operating range of the slider-crank mechanism is a function of the lengths of the crank, connecting the rod and the distance of the slider line from the crank pivot, therefore:

(6) $$\alpha_{max}=\pm {\text{ cos }}^{-1}\;\frac{d-l_{s}}{r}$$

**Figure 7:**
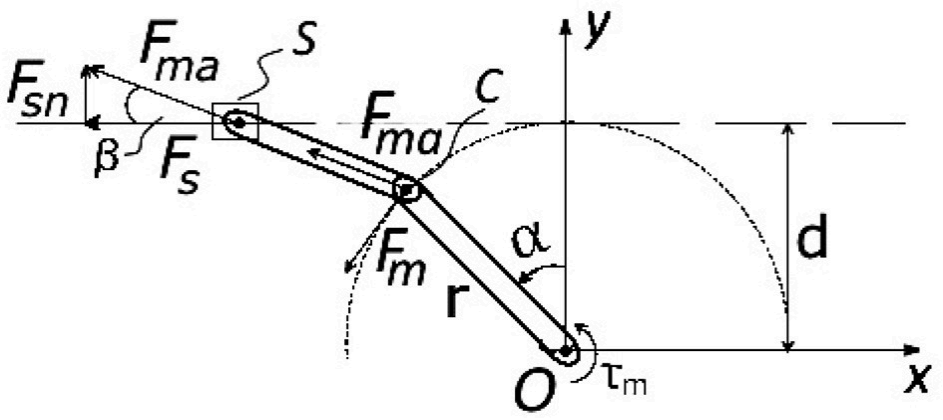
Sketch for slider-crank mechanism displaced on a motor side and used for the dynamics analysis.

In order to express the motor torque related to the force transmitted via PPC and later to the joint moment, static force analysis of the slider-crank mechanism is described below. The mass and inertia parameters of the connecting rod are neglected, while the inertia of the crank plate is taken into consideration. Figure 7 shows the force diagram of the slider-crank mechanism. The motor torque *M*_*m*_ is applied by the motor around the vector of the crank joint that is normal to the image plane and passes through the crank pivot point *O*. The applied momentum *M*_*m*_ is converted by the lever *r* to the force *F*_*m*_ at the other side of the crank link (point *C*).

The force *F*_*m*_ is equal to:

(7) $$\begin{array}{l} F_{m}=\frac{\tau_{m}}{r} \end{array}$$

The force *F*_*s*_ that acts on the push-pull cable end is represented by:

(8) Fs=Fma cos β

where *F*_*ma*_ – the axial force transmitted by the connected rod is expressed as:

(9) $$\begin{array}{l} F_{ma}=\frac{F_{m}}{\cos\left(\beta +\alpha \right)} \end{array}$$

### 2.4. Description of experiments

#### 2.4.1. Goals

Several experiments were performed in order to evaluate the transmission efficiency of the PPCs by comparing forces measured on the motor and respectively, the orthosis joint sides of the cable (*F*_*in*_ vs. *F*_*out*_). Based on these force measurements, Equations (4, 7–9) were used to calculate the torque required for the orthosis joints actuation.

The other point of interest during the experiments, was to observe the effect of velocity changes on operational and output forces.

#### 2.4.2. Experimental setup

For the experiments was used the test-stand device described in section 2.2. It was necessary to define several conditions related to the experimental setup, namely:

- PPC cable curvature is not restricted or guided in any way during actuation of the orthosis joints;
- During the experiments all 3 PPC cables remain connected to the orthosis joints and motors;
- The pulling phase is considered when the motor puts the cable in tension. The pushing phase is defined when the motor compresses the cable.

#### 2.4.3. Experimental protocol

For the experiments we considered actuating consecutively the hip and knee joints of the orthosis. The ankle joint was unactuated during all the experiments. However, the ankle PPC remained connected between the ankle joint and the motor. Actuation of the hip and the knee joints is realized by implying to the motor a sinewave of a constant amplitude. The amplitude is selected in such a way that the actuation in both directions is noticeable, therefore to illustrate the effect of the push and pull movements of the cable. The curvatures of PPCs are not restrained or guided in any specific way.

The force measurements are provided by the two force sensors (U9C), located on the joint (indicated in graphs by JF) and the motor sides (indicated in graphs by MF) of each PPC. This data was used later to determine the torque parameter τ_*m*_ with the expressions (4, 7–9).

The hip and knee joints were tested in separate trials. Therefore, two sets of experiments can be distinguished, for the hip and respectively, for the knee. Each set consisting of two tests with different motor velocities following a sinewave amplitude.

For the hip joint actuation experiments motor amplitude was set to ±25°. To vary the velocity, sinewave frequency was set to 0.15*Hz* and then to 0.05*Hz*.

For the knee joint experiments the motor amplitude was set to 30°, starting from the initial position when the orthosis leg is in vertical position. In contrast to the hip actuation experiments, hier PPC was always in tension due to the fact that the orhosis knee joint has limited range of motion and does not allow extension. Therefore, the dynamics of PPC in contraction was not characteristic for the knee actuation experiments. However, the same frequencies as the ones used in the hip experiments were used, 0.15*Hz* and then 0.05*Hz*.

#### 2.4.4. Data processing

Force and position measurements were collected from all the experiments and exported to MATLAB. Each experiment was performed in several trials. Out of continuous data stream an arbitrary period of 30 s was selected for further data processing. The same period was considered for all four experiments.

## 3. Results

### 3.1. Hip joint actuation, first set of experiments

**Figure 8:**
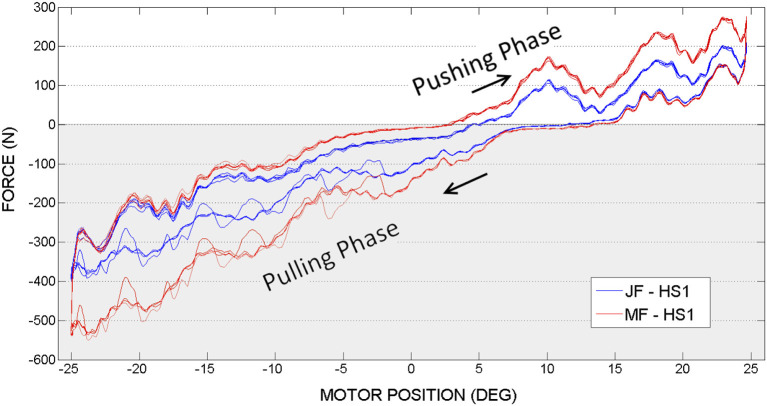
Hip actuation experiment, with frequency 0.15 Hz: Joint Force (JF) and Motor Force (MF) vs. Motor Position.

**Figure 9:**
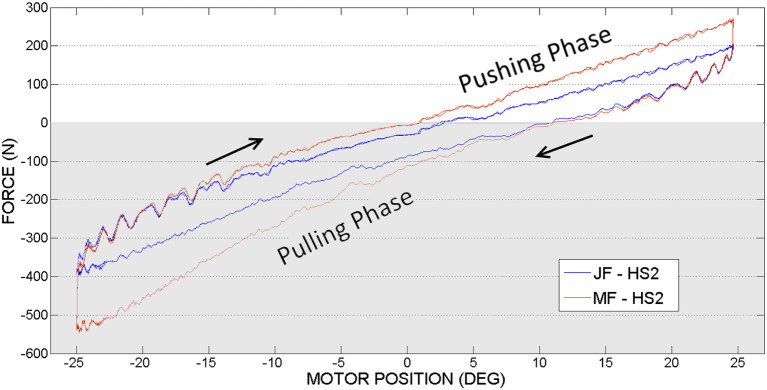
Hip actuation experiment, with frequency 0.05 Hz: Joint Force (JF) and Motor Force (MF) vs. Motor Position.

In Figures 8, 9 are illustrated the joint force (JF) and motor force (MF), measured by sensors located on both ends of the hip push-pull cable, function of the motor position angle. Two phases can therefore be distinguished in the figures, pulling (represented by a negative force on a graph with gray background) and pushing (represented by a positive force on a graph with white background). The transaction from pulling to pushing movement can be identified according to force readings. When the orthosis achieves the equilibrium position the force sensors register zero value. Pushing phase starts when the orthosis passes the equilibrium position and therefore sensors register positive readings. Considering that orthosis equilibrium position is not when the orthosis is perfectly vertical as the initial condition for the experiments, more pulling force is required to reach the desired amplitude (sinewave is offset from the equilibrium position).

**Figure 10:**
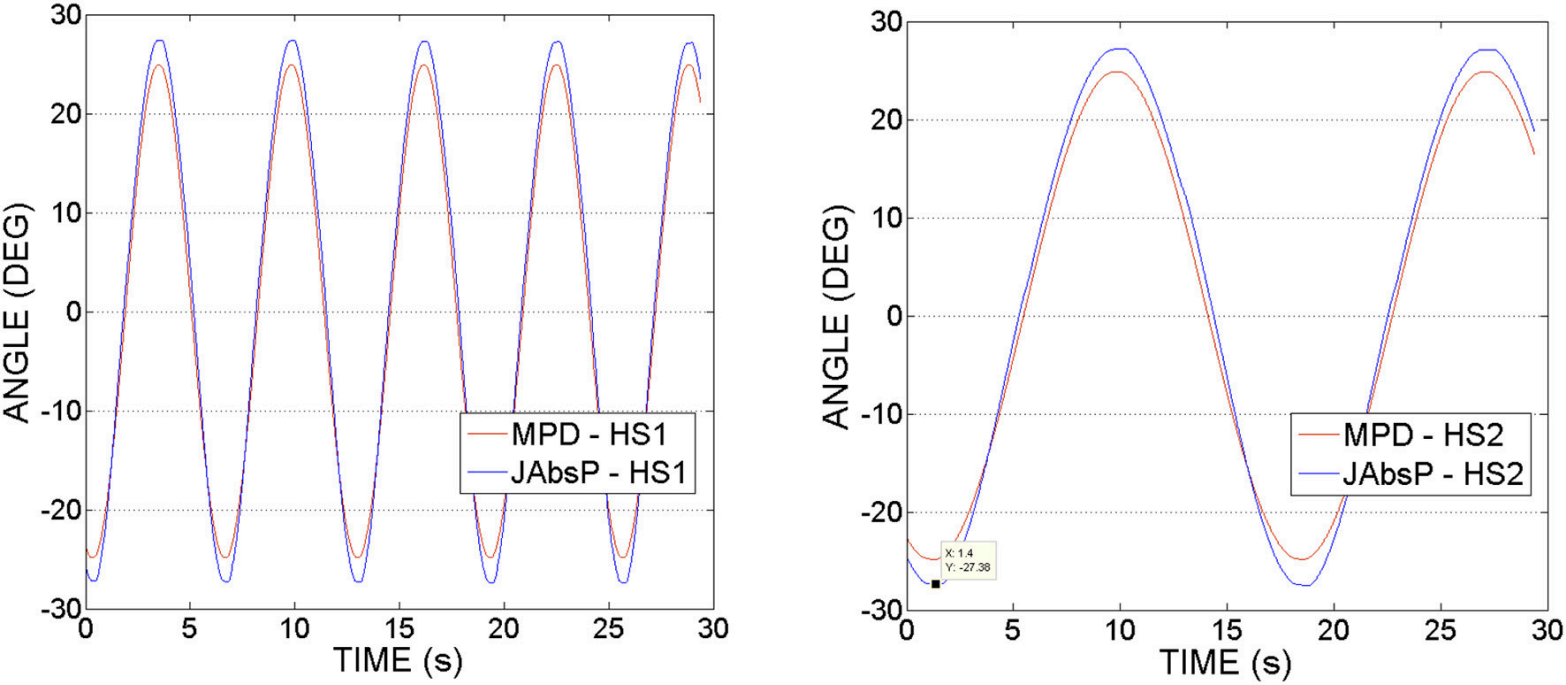
Hip actuation experiment, using frequencies of 0.15 and 0.05 Hz: Joint angular position (JAbsP on graph) and motor position (MPD) vs. Time.

Figure 10 shows joint (denoted by JAbsP on the graph) and motor angular positions(MPD) during the experiments with hip joint actuation. It can be noticed that the joint angle is following the output sinewave trajectory of the motor. These graphs demonstrate an appropriate position control without deviations from the imposed motion (no oscillations as have been seen in the force readings, from Figures 8, 9).

It can be also observed that the angle of the joint exceeds that of the motor by 2–5°. This appears due to the various geometrical dimensions of the slider-crank elements used on the joint and respectively, the motor sides, as specified in Table 2.

### 3.2. Knee joint actuation, second set of experiments

**Figure 11:**
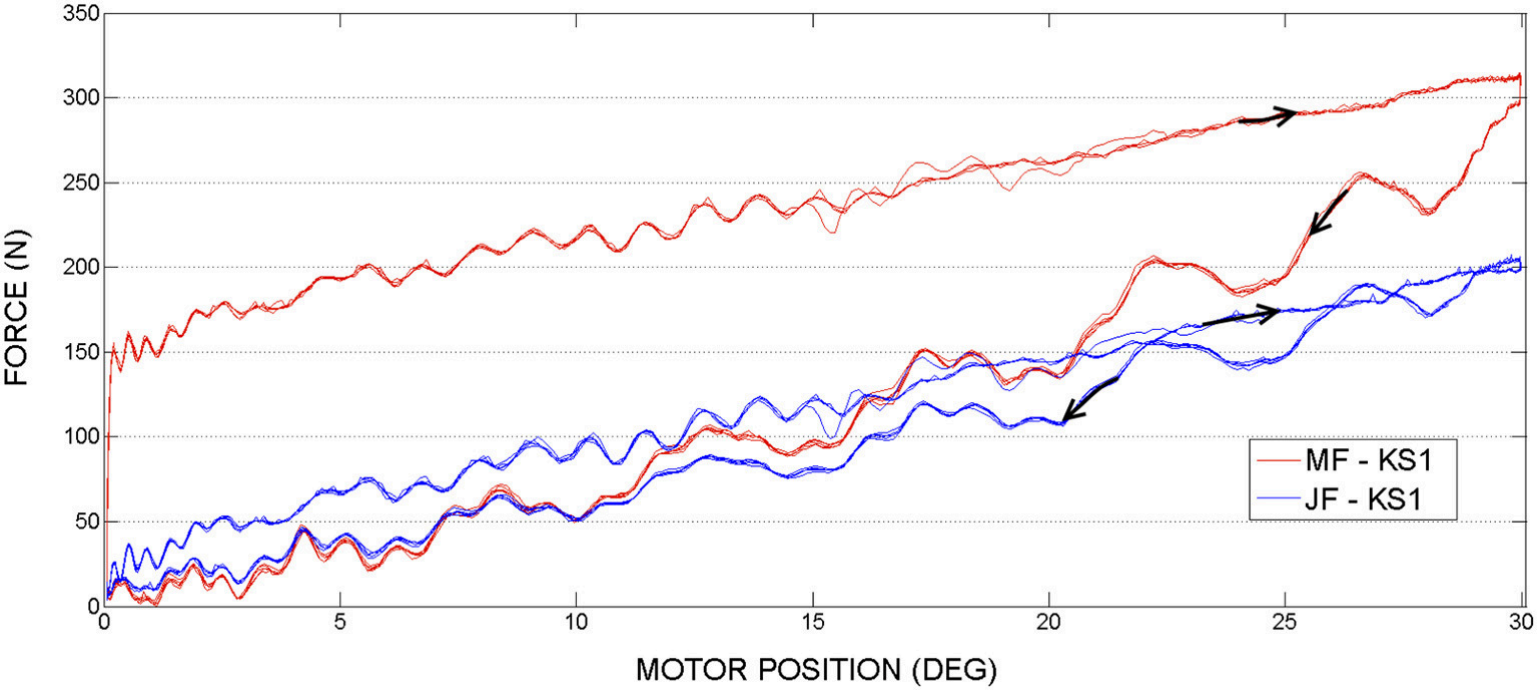
Knee actuation experiment, using frequency 0.15*Hz*: Joint Force and Motor Force vs. Motor Position.

**Figure 12:**
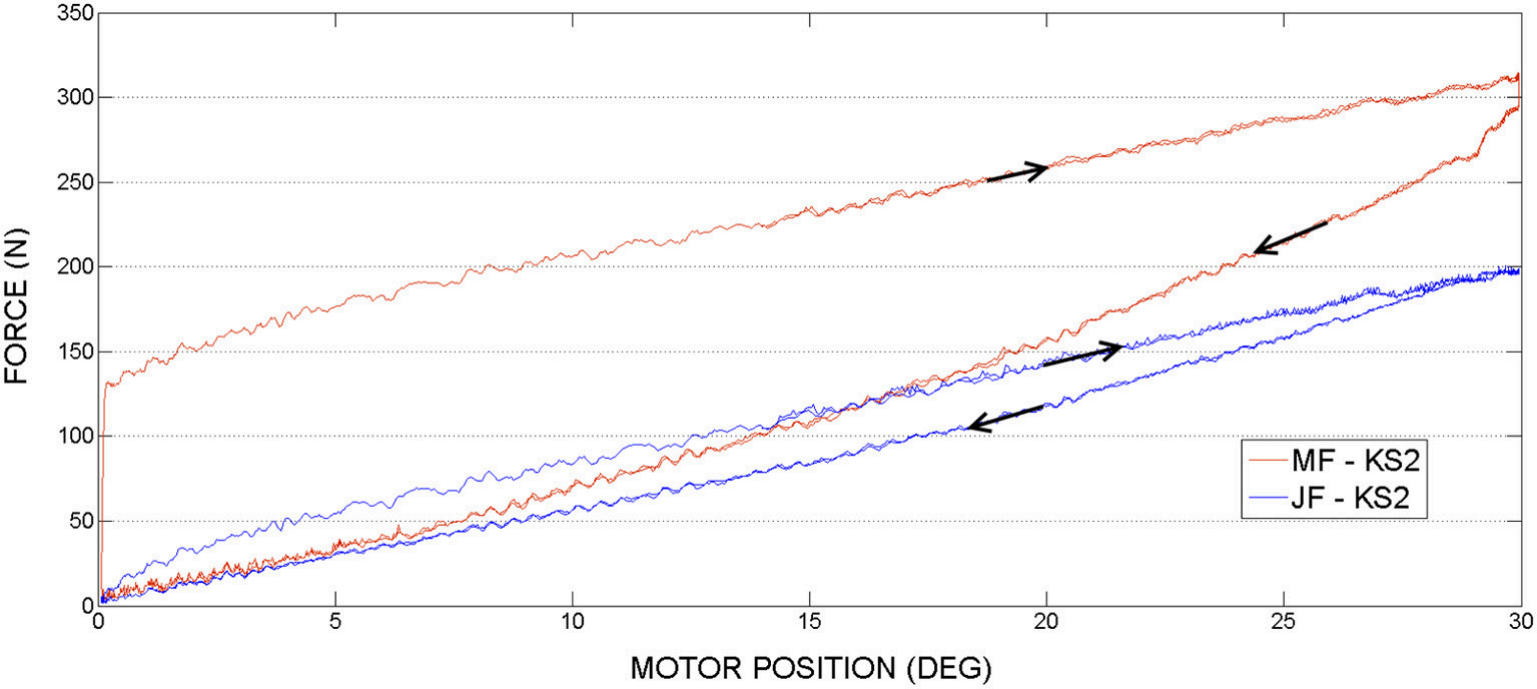
Knee actuation experiment, using frequency 0.05*Hz*: Joint Force (JF) and Motor Force (MF) vs. Motor Position.

In the second set of experiments, see Figures 11, 12 the knee joint motor has been actuated, while the hip and the ankle motors remained blocked.

**Figure 13:**
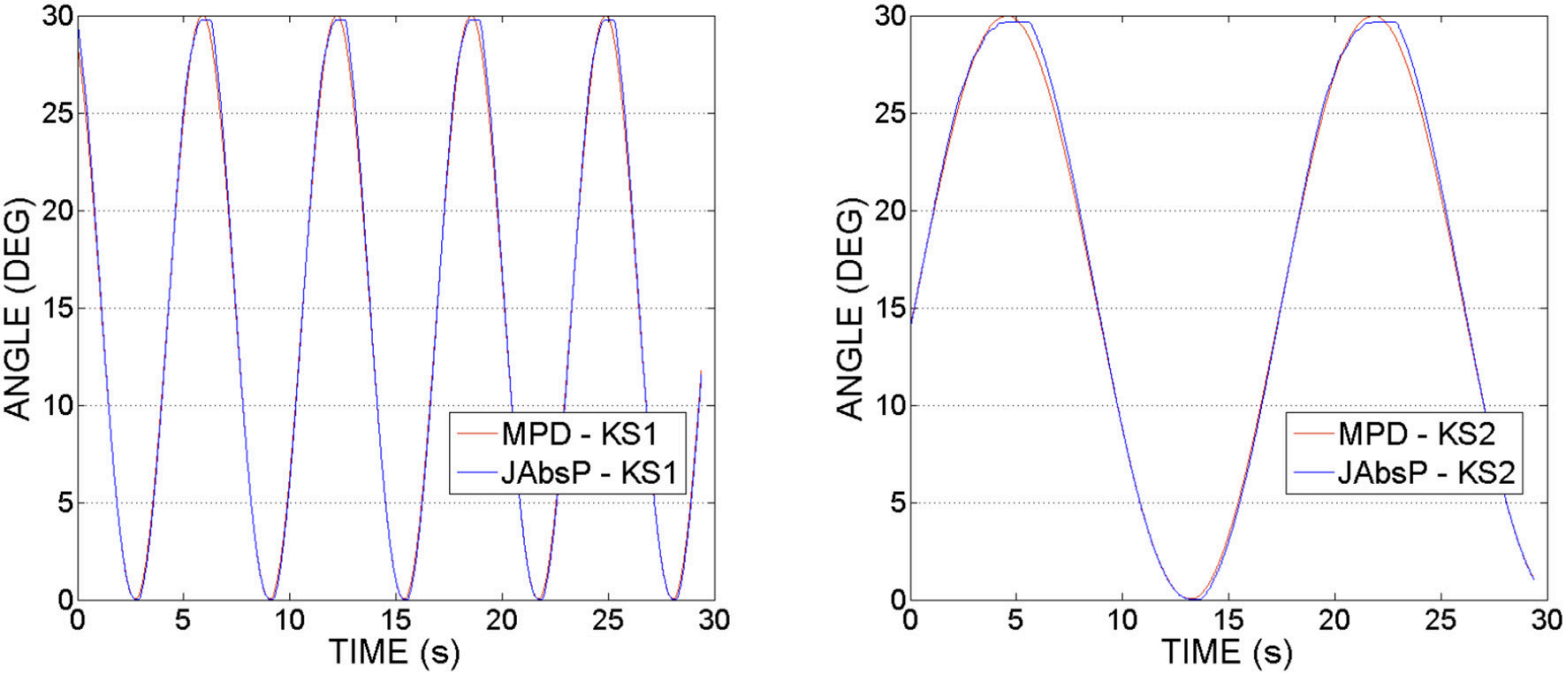
Knee actuation experiment, using frequencies of 0.15 Hz and, respectively 0.05 Hz: Joint angular position (JAbsP on graph) and motor position (MPD) vs. Time.

Figure 13 shows the joint (JAbsP on the graph) and motor angular positions (denoted by MPD on the graph) during the experiments with the knee joint actuation. We can see the output sinewave trajectory of the motor of 30° and the output angle of the joint. The joint closely follows the trajectory imposed by the motor.

**Figure 14:**
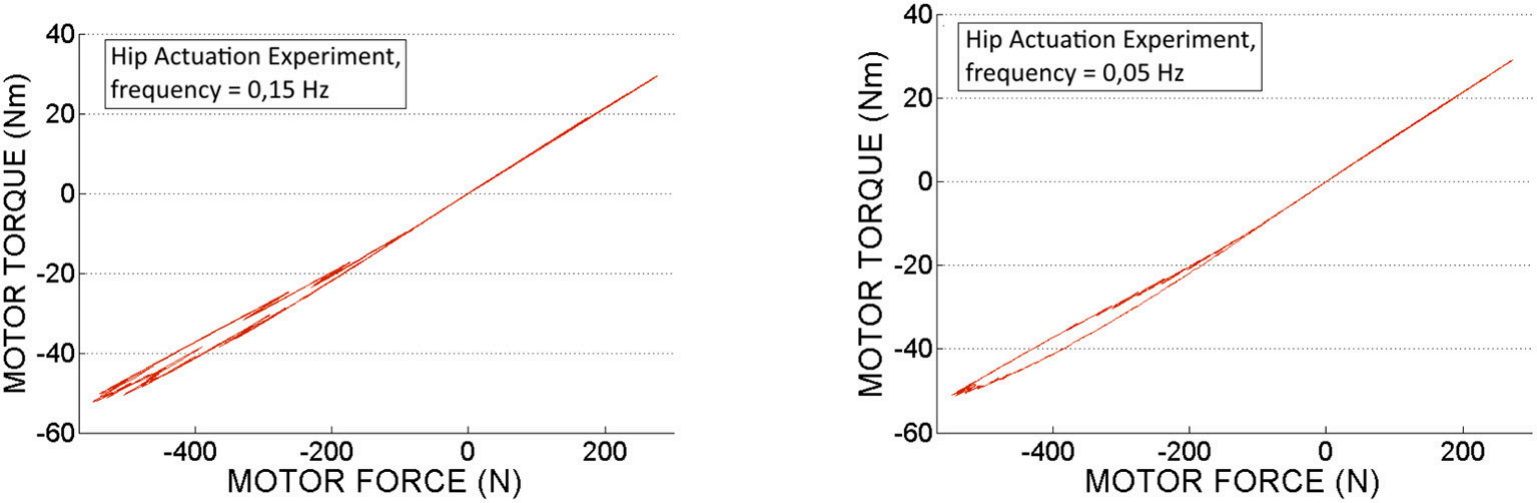
Motor torque calculated based on experimental motor force data, obtained from the *hip actuation experiments*.

**Figure 15:**
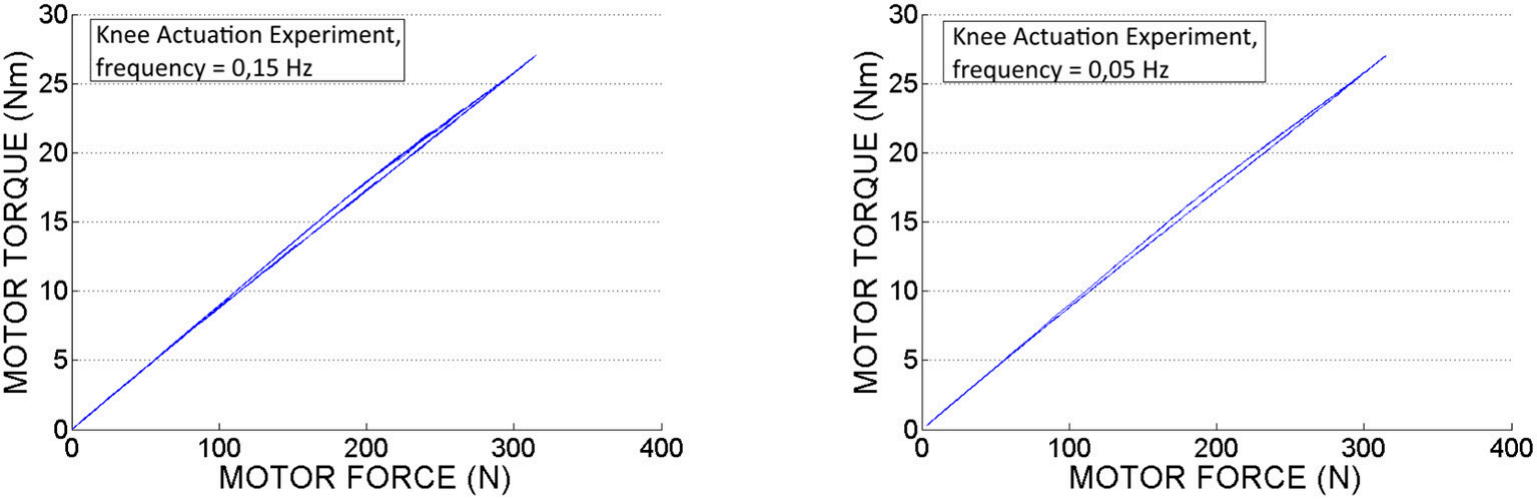
Motor torque calculated based on experimental motor force data, obtained from the *knee actuation experiments*.

In Figures 14, 15 we can see the calculated motor torque function of input force *F*_*in*_, obtained from the hip and knee actuation sets of experiments. According to the graphs we can observe almost linear relation between motor torques and forces.

In Table 3 are displayed push-pull cables efficiencies calculated based on peak forces (*F*_*in*_ and *F*_*out*_ ) measured in the experiments. According to this data we can conclude that the difference between the efficiencies values is not considerable for the two selected speeds. Also, it can be observed that in pushing phase the efficiency is slightly higher as less force is required to perform the movement. Therefore, the efficiency depends on the operating loads, which is in line with the specifications from literature, presented in the introduction part of this article. It can be also observed that in contrast to the hip PPC, the knee PPC is operating with less efficiency. This can be explained by the different geometry and of the knee cable, which is longer and thinner with an increased bending curvature.

**Table 3:** Hip and knee push-pull cables efficiencies during pushing and pulling phases, measured at the maximum force magnitudes.

**Efficiency**	**0.15 *Hz***	**0.05 *Hz***
Hip joint PPC (Pulling–Pushing)	71–76%	73-74%
Knee joint PPC (Pulling)	65%	64%

## 4. Discussion

In this paper were addressed the critical points of cable-driven transmissions, including their dynamic behavior, main advantages, and drawbacks with the focus on the push-pull cable configuration. PPC-based transmission has been implemented in actuation of a new gait rehabilitation robot CORBYS, but in order to test the performance of actuation system its simplified version (a test-stand) has been used for the experiments. One of the main goals of experiments was to estimate the transmission efficiency of the PPC and to observe the overall dynamic behavior of the system. The obtained results suggest to think about following control optimization strategies for compensation of friction, nonlinearities, and backlash issues.

All types of cable-conduit transmissions present drawbacks, such as nonlinear friction highly dependent on curvature and geometry of the cable. The dynamic behavior and functional characteristics of Bowden and PPC transmissions are very similar, but there are also few differences. In comparison to PPC, Bowden cable-based actuation systems can operate only in pulling direction. Taking into account that PPCs are larger in diameter and more stiff, they are able to transmit larger forces compared to the Bowden cables. Moreover, PPCs have the ability to transfer forces in two directions, pushing and pulling.

Considering hip joint experiments, if we compare force readings provided by the motor side force cell (MF on a graph, in RED) and joint force cell (JF on a graph, denoted in BLUE), it can be observed that the higher force is needed at the motor side. This outcome appears due to the energy losses caused by nonlinear friction along PPC actuation mechanism.

When the motor achieves the maximum imposed angle position (−25° or 25°) it changes the actuation direction. Starting from that moment, according to the graphs, it can be observed that the gravity helps the movement and JF and MF are overlapping. According to the experimental results illustrated in Figures 8, 9, in limits of the selected frequencies, the required force magnitudes are similar. However, for higher velocities, due to the dynamics of PPC, the force oscillations are more evident. Considering these oscillations we can state that the PPC performs better in tension.

In contrast to the hip actuation experiments, the push-pull cable in knee joint experiments was always in tension because the orthosis leg was not passing through the equilibrium position. Therefore, observing the PPC dynamics while in contraction (which is pushing phase) is missing for the knee actuation experiments. The difference in force readings between the motor sensor (MF) and joint sensor (JF) can be visualized on the graphs. This difference is produced by losses of energy due to the dynamics of the actuation system and push-pull cable nonlinear friction. This force difference is more evident in contrast to the hip PPC, due to the physical characteristics of the PPC used for the kne actuation joint. Namely, it is longer, thinner (see Table 1) and was changing cable curvature due to the orthosis motion. Similar to the hip joint actuation set of experiments, selected velocities do not have any considerable effect on force output magnitudes.

Taking into account that the exact model of the PRL+ actuator is difficult to obtain due to its nonlinear characteristics, the motor torque τ_*m*_ was determined based on sensors force measurements (MF) and using formulas 4, 7–9 presented in theoretical part of section 2.

The establishing of the motor torque τ_*m*_ was one of the goals followed in this contribution. This torque will be used as a reference for further investigations on torque control strategies as an alternative to the position control used so far. Torque control is especially important for the applications where human-robot interaction is involved, such as in exoskeleton applications. This solution permits to avoid integration of the expensive torque sensors in the system and reduces its mechanical design complexity.

## 5. Conclusion

Cable-based actuation systems show many advantages over classical actuators when implemented in various robotics related applications. The employment of this type of actuation in wearable devices permits the dislocation of the actuators from the orthosis so that inertia of the motors is not imposed on human body, therefore, improving safety and functional aspects. Still, issues related to control challenges, specific mechanical design requirements and assumptions have to be considered. These aspects were addressed in this paper together with the evaluation of cable-based force transmission system for powered orthoses.

Several experiments were performed to evaluate the transmission efficiency of the PPCs by comparing forces measured on the motor and respectively, the orthosis joint sides of the cable. Based on these force measurements, the torque required for the orthosis joints actuation was computed.

Additionally, we noticed that the efficiency of the PPC force transmission is highly dependent on the configuration of the actuation components of the system, such as mechanical design, geometry of the PPC, bending angles, and cable preloads.

The other objective during the experiments, was to observe the effect of velocity changes on operational and output forces. According to obtained results the force amplitude does not change considerably but the force oscillations are more evident at higher velocities.

According to the obtained results, can be concluded that all specific features and compromises typical for cable-conduit-based transmissions are also characteristic to PPC actuation. Still, using PPC actuation in certain applications could be preferred due to the number of individual advantages. For example, capability to transfer larger forces in two directions and less complex mechanical construction of the actuation system. The use of PPC actuation systems is advised in applications where the light-weight design and transmission of large forces are required, definitely a solution to consider in wearable devices.

## Author contributions

SG, CR-G, and VG were working together on experimental setup and post-processing of results. BV and DL were providing guidance in this research study, as well they contributed in a writing process of this article.

### Conflict of interest statement

The authors declare that the research was conducted in the absence of any commercial or financial relationships that could be construed as a potential conflict of interest.
